# Noninferiority of ^99m^Tc-Ethylenedicysteine-Glucosamine as an Alternative Analogue to ^18^F-Fluorodeoxyglucose in the Detection and Staging of Non-Small Cell Lung Cancer

**DOI:** 10.1155/2018/8969714

**Published:** 2018-03-15

**Authors:** Dong Dai, F. David Rollo, Jerry Bryant, E. Edmund Kim

**Affiliations:** ^1^Tianjin Medical University Cancer Institute and Hospital, Tianjin, China; ^2^Cell*➤*Point Biotechnology, Centennial, CO, USA; ^3^University of Texas MD Anderson Cancer Center, Houston, TX, USA; ^4^University of California, Irvine, CA, USA

## Abstract

*Objective.* ^99m^Tc-ethylenedicysteine-glucosamine (^99m^Tc-EC-G) was developed as a potential alternative to ^18^F-FDG for cancer imaging. A Phase 2 study was conducted to compare ^18^F-FDG PET/CT and ^99m^Tc-EC-G SPECT/CT in the detection and staging of patients with non-small cell lung cancer (NSCLC). This study was aimed to demonstrate that ^99m^Tc-EC-G SPECT/CT was not inferior to ^18^F-FDG PET/CT in patients with confirmed NSCLC.* Methods.* Seventeen patients with biopsy proven NSCLC were imaged with ^99m^Tc-EC-G and ^18^F-FDG to detect and stage their cancers. Imaging with PET/CT began 45–60 minutes after injection of ^18^F-FDG. Imaging with ^99m^Tc-EC-G began at two hours after injection (for 5 patients) or three hours (for 12 patients). SPECT/CT imaging devices from the three major vendors of SPECT/CT systems were used at 6 participating study sites. The image sets were blinded to all clinical information and interpreted by independent PET and SPECT expert readers at a central independent core laboratory.* Results.* 100% concordance between ^99m^Tc-EC-G and ^18^F-FDG for primary lesion detection, lesion location and size, and confidence that the biopsied lesion was malignant. There was 70% agreement between ^99m^Tc-EC-G and ^18^F-FDG for metastatic lesion detection, location and size, and confidence that the suspicious lesions were malignant.* Conclusions.* Evaluation of primary and suspicious metastatic lesions detected by ^99m^Tc-EC-G and ^18^F-FDG on 17 patients resulted in excellent agreement for detection of primary and metastatic lesions. The study results indicated that ^99m^Tc-EC-G SPECT/CT has the potential to be a clinically viable alternative to ^18^F-FDG PET/CT and ^99m^Tc-EC-G is not inferior to ^18^F-FDG PET/CT.

## 1. Introduction

### 1.1. Molecular Mechanism of Ethylenedicysteine-Glucosamine (EC-G) in Oncology

**Figure 1:**
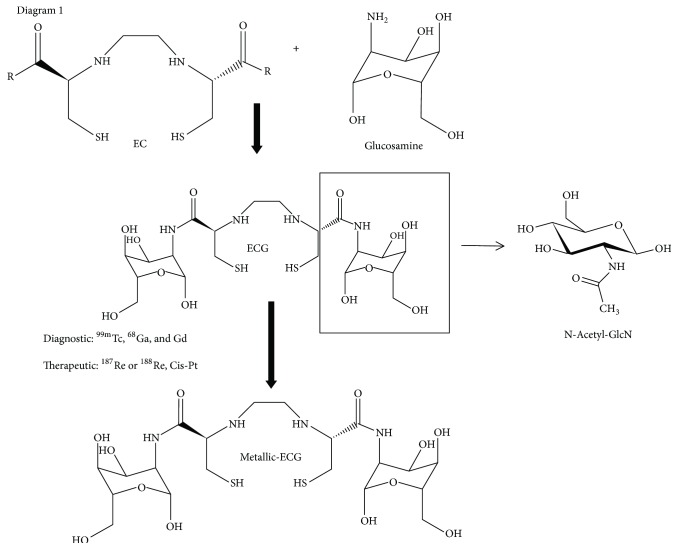
Structure of ethylenedicysteine-glucosamine (EC-G) and relationship to N-acetylglucosamine (GlcNAc).

^18^F-Fluoro-2-deoxyglucose (FDG), a nonmetabolizable 2-deoxyglucose analogue, blocks glycolysis and inhibits protein glycosylation [1]. Increased ^18^F-FDG uptake is used to visualize cancer cells in patients using PET/CT [2, 3]. In addition, it is known that cell uptake of glucose and glutamine is directed by growth factor signaling. Both glucose and glutamine are involved in mitochondrial tricarboxylic acid (TCA) cycle integrity, glycolysis, ATP production, and glycosylation [4, 5]. For example, glutamine is a known precursor amino acid for the synthesis of O-linked-Beta N-acetylglucosamine (GlcNAc), one of the main initiators of the Hexosamine Biosynthetic Pathway (HBP) [6–8]. Glutamine combines with fructose-6-phosphate from the glucose glycolytic pathway in the presence of glutamine fructose-6-phosphate transferase (GFAT), an initiating enzyme, resulting in the synthesis of glucosamine-6-phosphate [7, 8]. A series of enzymatic steps result in the production of uridine diphosphate-N-acetylglucosamine (UDP-GlcNAc) in endoplasmic reticulum (ER). The synthesized UDP-GlcNAc is transported from ER to the Golgi apparatus via the UDP-GlcNAc transporters and is then utilized as a donor substrate for the* N*- and* O*-linked glycosylation of extracellular and membrane proteins [9, 10]. O-linked glycosylation is regulated by the terminating enzyme O-linked GlcNAc transferase (OGT). OGT is the enzyme responsible for the addition of a single N-acetylglucosamine (GlcNAc) residue to the hydroxyl groups of serine and/or threonine residues of target proteins. The hexosamine signaling pathway terminating in O-linked GlcNAc cycling has been shown to be involved in cellular signaling cascades and regulation of transcription factors involved in cancer biology and is a requirement for cell membrane and protein synthesis for cell regeneration in other tissue types as well [10–12]. GlcNAc, a glucose analogue, can be taken up by cells through glucose transporters [13, 14]. In cancer cells, GlcNAc glycosylation has been shown to play a role of angiogenesis and metastasis [12]. We have then developed a metabolic agent that mimics GlcNAc pathway by combining the chelator, ethylenedicysteine (EC), to two molecules of D-glucosamine [15–17] creating the chemical analogue ethylenedicysteine-glucosamine (EC-G), a metabolic conjugate compound containing two molecules of GlcNAc [18]. The molecular mechanism of EC-G involves trapping by phosphorylation, docking by UDP conjugation at position-1 of glucosamine, fusing by recruiting NfKb protein conjugation at position-1 of glucosamine, and translocation of fused glycoprotein to cell nuclei to regulate Sp1 and myc [19–21]. In addition to its application in molecular diagnostic imaging, EC-G binds to therapeutic metals that can be used to treat cancer cells (Figure 1).

### 1.2. Cellular Uptake Differences between ^99m^Tc-EC-G and ^18^F-FDG

**Figure 2:**
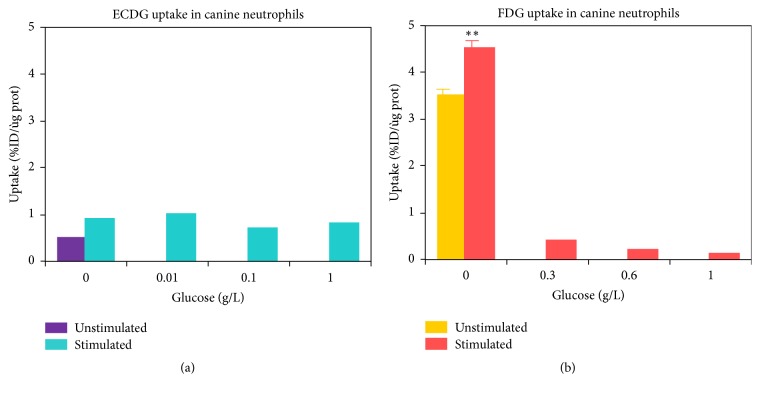
Comparative uptake of ^99m^Tc-EC-G versus ^18^F-FDG in stimulated neutrophils as a function of glucose concentration (Courtesy of Alexis Broisat and Dr. David K. Glover: Department of Medicine, Cardiovascular Division, University of Virginia, Charlottesville, VA). *∗∗* indicates a significant uptake difference (*P* < 0.05,* t*-test) between glucose loading and control groups.

**Figure 3:**
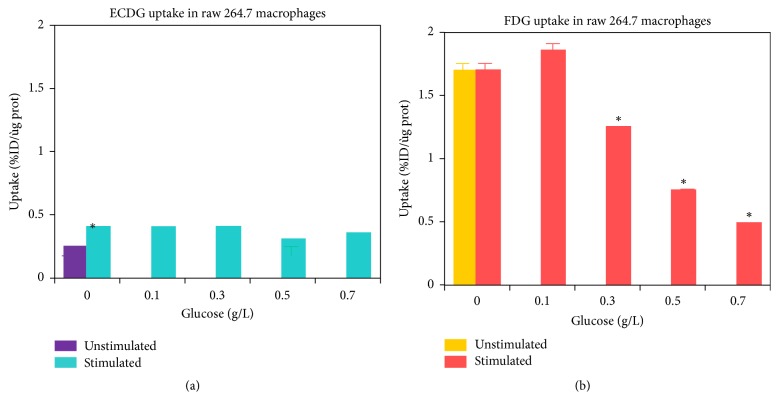
Comparative uptake of ^99m^Tc-EC-G versus ^18^F-FDG in stimulated macrophages as a function of glucose concentration (Courtesy of Alexis Broisat and Dr. David K. Glover: Department of Medicine, Cardiovascular Division, University of Virginia, Charlottesville, VA). *∗* indicates a significant uptake difference (*P* < 0.05,* t*-test) between glucose loading and control groups.

It is well known that ^18^F-FDG localizes in inflammatory cells [14] and infection [15]. Glucose loading studies were performed with stimulated macrophages and neutrophils for both ^99m^Tc-EC-G and ^18^F-FDG (Figures 2 and 3). It can be seen that increasing the concentration of glucose in the presence of macrophages or neutrophils results in a decrease in ^18^F-FDG uptake due to competition between ^18^F-FDG and glucose. Alternatively, ^99m^Tc-EC-G uptake in macrophages or neutrophils is minimal and remains constant independent of the amount of glucose loading. This suggests the potential for ^99m^Tc-EC-G to have increased diagnostic accuracy over ^18^F-FDG in patients having an infectious process such as tuberculosis, pneumonia, or a granulomatous disease. ^99m^Tc-EC-G should have a clear advantage compared to ^18^F-FDG in evaluating the efficacy of therapy in real time while the patient is undergoing therapy. The reason is the inflammatory reaction of the collateral tissue surrounding the tumor following the initiation of chemotherapy or radiation therapy. Consequently, an ^18^F-FDG PET/CT imaging procedure is not able to differentiate between the inflammation and the impact of the therapy on the tumor because the tumor is obfuscated by the presence of the inflammation.

^99m^Tc-EC-G localizes in the nucleus of cells. Several experiments, which were proof of concept studies, demonstrated intranuclear localization that was validated by performing cytosol studies as well as thymidine incorporation studies [13]. The results of cytosol study showed ^18^F-FDG to be localized completely within the cytoplasm whereas the ^99m^Tc-EC-G localized in the nucleus. The thymidine incorporation studies showed that glucose and ^99m^Tc-EC-G localize in all three phases of cell proliferation (G0/G1, G2/M, and S), while there was no uptake of ^18^F-FDG in any of the phases of cell proliferation [13]. This suggests that EC-G (GlcNAc) and glucose are both involved in the proliferation/growth activity of the cells, whereas ^18^F-FDG is not in glycosylation process. Lack of ^18^F-FDG activity in the proliferation and growth studies is attributed to the location of the fluorine atom in position 2 of the molecule, which prevents acetylation occurrence and is recognized by UDP and participation in cell proliferation and growth.

### 1.3. Metallic Application with EC-G

Using a chelator such as EC, radiodiagnostic (^99m^Tc, ^111^In, and ^68^Ga), radiotherapeutic (^177^Lu, ^188^Re, ^90^Y, ^225^Ac, and ^223^Ra), and nonradioactive metals (^187^Re, Pt, and Cu) have the opportunities to be incorporated. Thus, for both diagnostic and therapeutic metallic labels, EC-G is considered as a new theranostic molecule.

### 1.4. ^99m^Tc-EC-G Characteristics in Oncology (Phase 1 Finding)

**Figure 4:**
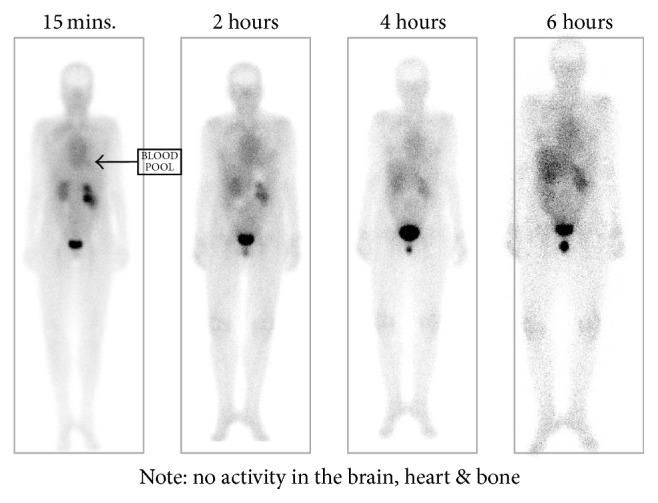
Whole-body planar biodistribution images of ^99m^Tc-EC-G at 15, 30, 60, 120, and 240 minutes after injection. Note absence of activity in the brain, heart, and bone.

Planar biodistribution images of ^99m^Tc-EC-G (Figure 4) from our Phase 1 study are hallmarked by no uptake in the normalized bone, brain, or myocardium. In addition, there is minimal uptake in the liver until late in the imaging cycle. This demonstrates a different biodistribution pattern compared to ^18^F-FDG, which has significant uptake in the normalized heart, brain, and bone marrow. It is postulated that the absence of ^99m^Tc-EC-G uptake in the normal brain is due to the charge on the EC-G which prevents the compound from crossing the blood-brain barrier. However, in the presence of a tumor lesion, the uptake in the region(s) of abnormality should occur. Thus, a ^99m^Tc-EC-G whole-body scan, which would include the brain, could potentially be used to determine if brain metastasis is present. To extend evidence of ^99m^Tc-EC-DG imaging efficacy in identifying anatomical regions and in determining extent of disease in these patients as compared to ^18^F-FDG PET/CT imaging, here, we report a multicenter Phase 2 study comparing ^99m^Tc-EC-G SPECT/CT with ^18^F-FDG PET/CT in patients with non-small cell lung cancer (NSCLC).

## 2. Materials and Methods

### 2.1. Primary Endpoint and Objectives

CGMP grade EC-G was manufactured at J-STAR Research, Inc. (South Plainfield, New Jersey). ^99m^Tc-EC-G was delivered by the local radiopharmacy to the clinical trial site. The endpoints for this Phase 2 study were as follows:

1. Noninferior to the sensitivity of ^99m^Tc-EC-G SPECT/CT versus ^18^F-FDG PET/CT for the biopsied primary tumor.
2. Noninferior to the detectability and confidence level for determining malignancy/or not for metastatic disease using ^99m^Tc-EC-G SPECT/CT versus ^18^F-FDG PET/CT.

The Phase 2 trial was a multicenter study conducted on a total of 22 untreated patients (12 women and 10 men) aged 46.4 to 89.5 years (mean age: 68.5), who had nonincisional biopsy-definitive evidence of NSCLC or cytology results confirming NSCLC from a bronchoscope procedure. Patients underwent imaging procedures with both ^99m^Tc-EC-G SPECT/CT and ^18^F-FDG PET/CT. Of the 22 enrolled patients, 17 patients completed the study.

Within 7 days of qualification for the study or during prestudy procedures, ^18^F-FDG was administered and a PET/CT scan was performed. A dose range of 370 to 740 MBq of ^18^F-FDG was injected into a peripheral vein of the upper extremity (actual dose administered was to be consistent with the recommendations of the vendor of the PET/CT system). A CT scan without contrast from the base of the skull to the upper thigh level was acquired 60 minutes following injection of the ^18^F-FDG. This was used for attenuation correction (AC). Alternatively, a High Quality CT Scan (HQS) sufficient to provide an AC map and adequate for anatomical localization when fused with the PET image could have been substituted for the attenuation correction CT scan. Whole-body PET imaging from the base of the skull to the upper thigh level was immediately obtained after the attenuation correction CT scan or the alternative HQS. If the HQS alternative was not utilized, the PET scan was followed by a diagnostic CT scan (without contrast from base of skull to upper thigh level). This CT scan was used for anatomical localization. Standard practice imaging protocols were used unless otherwise specified or clinically indicated.

^99m^Tc-EC-G was administered and a SPECT/CT scan performed from 1 to 3 days following the PET/CT scan (or within 45 days if the PET/CT scan was done as a part of prestudy procedures). A target activity level of 925 MBq (range from 740 to 1110 MBq) of ^99m^Tc-EC-G was administered through an indwelling IV line. The dose of EC-G was approximately 5 mg for the first 5 enrolled patients and approximately 1 mg or less for the remaining 12 patients. A HQS without contrast from the base of the skull to the upper thigh level (covering the same field of view as the PET/CT scan) was acquired approximately 2 hours following injection of 5 mg of ^99m^Tc-EC-G and at approximately 3 hours following injection of the 1 mg (or less) dose of ^99m^Tc-EC-G. The CT information was used to create an attenuation correction map for the attenuation correction, whereas the HQS was fused with the SPECT images to provide anatomical localization of lesions. Immediately after the HQS was performed, the patient was given a SPECT scan from the base of the skull to the upper thigh level. Standard practice imaging protocols were used unless otherwise specified or clinically indicated.

After the last imaging procedure, a 21-day follow-up was performed (without a patient visit) to acquire any additional imaging, surgical/pathology tissue diagnostic results, and treatment documentation. Safety was evaluated by assessing vital signs, EKGs, physical examinations, clinical laboratory test results, and the incidence and severity of AEs.

## 3. Statistical Considerations

### 3.1. Overview

The Phase 2 study was designed to be exploratory and include up to 25 patients with a biopsy (or cytology report from a bronchoscope) definitive diagnosis for NSCLC. The study design included 7 centers with no more than 8 patients completing the entire protocol at any center. This number of patients was considered sufficient to obtain the necessary data and knowledge to document continued safe use of ^99m^Tc-EC-G in multiple centers and to organize and plan a Phase 3 study.

### 3.2. Core Lab Interpretation Process: Independent Reading

The endpoints for this study include estimates of agreement (based on anatomical location and measures of extent of disease (staging) above and below the diaphragm) between PET/CT and SPECT/CT. The first endpoint was ^99m^Tc-EC-G SPECT/CT not being inferior to ^18^F-FDG PET/CT based on tissue specific pathology results for the primary lesion. In terms of the primary lesion, tissue specimens with a pathological diagnosis of malignancy were classified as positive. Any other pathology results, such as benign or inflammatory conditions, were classified as negative. The second endpoint was ^99m^Tc-EC-G SPECT/CT not being inferior to ^18^F-FDG PET/CT in the detection of suspicious malignant lesions (metastasis). Measures were based on detectability of suspicious lesions in one or more of the following anatomical zones: lobes for the right and left lungs; the mediastinum; the liver; and the adrenal glands.

A five-page interpretation form was completed by each core laboratory independent reader for each image set performed on each patient. The image interpretation was based on an objective score 1–5 with 5 representing the highest positive score, where the reader evaluated the following factors:

  Diagnostic image quality above and below the diaphragm
  Detectability of primary lesion and confidence that it represents cancer
  Detectability of any suspicious metastatic lesions and the anatomical zone for the lesion as well as the confidence that the lesion represents a malignancy
  Lesion size, scored as a range (1: <5 mm; 2: 5 mm–1 cm; 3: >1 cm)

The SPECT/CT image sets were read and interpreted by two dedicated independent SPECT specialists. Separately, the PET/CT image sets were read and interpreted by two dedicated independent PET specialists. All reads were blinded to clinical information other than knowing all patients enrolled had positive tissue diagnosis of NSCLC for the primary lesion.

When PET and/or SPECT readers at the core laboratory identified suspicious lesions beyond the primary (biopsied) lesion, they scored lesion detectability, identified the anatomical location and lesion size, and recorded their confidence that the lesion(s) was (were) malignant. When more than one lesion was identified in a given anatomical zone, the reader was instructed to evaluate the single lesion they felt best represented the diagnosis for the group. The objective results were provided to the biostatistician to perform the noninferiority analysis comparing the ^99m^Tc-EC-G SPECT/CT results to the ^18^FDG PET/CT results for detecting primary as well as metastatic lesions on patients who had a confirmed biopsy proven diagnosis of NSCLC.

## 4. Results

### 4.1. Safety

All AEs that were reported during the study occurred after the administration of ^99m^Tc-EC-G. All but 2 AEs, nausea and vomiting, were considered unrelated to the study drug. These 2 AEs for nausea and vomiting occurred in 1 patient after administration of ^99m^Tc-EC-G and were classified as mild and unlikely related to the study drug. Most AEs were classified as mild or moderate, with 3 AEs (international normalized ratio increased, hypokalemia, and embolism) in 2 patients being classified as severe. There were 6 SAEs (obstructive pneumonia, hypokalemia, international normalized ratio increased to 2 times the upper limit of normal, ataxia, speech impairment, and embolism) in 3 patients. All moderate and severe AEs were evaluated and deemed by the Safety Panel to be unrelated to the study drug. Overall, ^99m^Tc-EC-G and ^18^F-FDG were well tolerated.

### 4.2. Imaging Results

#### 4.2.1. Primary Lesion

The ^18^FDG PET/CT and ^99m^Tc-EC-G SPECT/CT scan results showed 100% concordance for detection of primary lesions, determination of lesion size, and confidence that the detected lesion was malignant. The detection of primary lesions was not influenced by the dose of ^99m^Tc-EC-G or by the time of imaging in the range of doses and times used in the 2 studies. The detection of the primary lesion was also not influenced by the type of imaging device or the device vendor (imaging devices from the three major international vendors were used in the study). Overall, the study results demonstrated noninferior detection of primary lesions (biopsied) of ^99m^Tc-EC-G SPECT/CT compared to ^18^F-FDG PET/CT (sensitivity only). The reader's average score [1–5] for detectability, lesion location and lesion size, and confidence that the lesion was malignant averaged 4.6 for the PET readers and 4.5 for the SPECT readers.

There was 1 patient who entered the Phase 2 study with a negative biopsy for NSCLC. However, this patient smoked, showed all of the clinical symptoms for lung cancer (e.g., blood in sputum), and had diagnostic findings on the CT scan that the radiologist felt were consistent with a diagnosis of a lung malignancy. The radiologist recommended that the referring physician order a ^18^F-FDG PET/CT scan. The decision was made to also perform a ^99m^Tc-EC-G SPECT/CT scan which also showed a positive finding. Based on the positive results of the ^18^F-FDG PET/CT scan, the patient was sent to surgery for tumor removal, and the extracted tumor was found positive for NSCLC. In this case, the use of ^18^F-FDG PET/CT and separately ^99m^Tc-EC-G SPECT/CT scanning both achieved an accurate diagnosis even in light of the original negative biopsy. This example shows the value of oncology imaging to assure patients receive the correct diagnosis and therefore the most appropriate therapy.

#### 4.2.2. Metastatic Lesions

Metastatic lesions were also shown to localize ^99m^Tc-EC-G for both the 1 and 5 mg dose of EC-G. However, the detectability of some lesions in dense tissue such as the liver and adrenal glands was shown to be affected by the characteristics of the SPECT/CT system used. Specifically, lesion detectability with select SPECT/CT systems was at a lower confidence level compared to PET/CT. This occurred on two SPECT/CT systems where the slice thickness used to reconstruct the AC map and fuse the SPECT and CT images was 1 cm. Unfortunately, these two devices accounted for all reported lesions in the liver (3 lesions) and adrenal glands (1 lesion). This occurred because the 1 cm slice thickness used for the reconstruction of the AC maps resulted in artifacts and distortion of the SPECT/CT fused image. This of course impacted image quality and detectability of small lesions. Despite this issue, there was 70% overall agreement between SPECT/CT and PET/CT for all metastatic lesions. There was 83% agreement for metastatic lesions detected in the lungs (10/12 lesions), 75% agreement for lesions in the mediastinum (3/4 lesions), 66% agreement for liver lesions (2/3), and 0% agreement for adrenal lesions (0/1). Retrospectively, it was reported that the suspicious adrenal lesion was a midline abdominal lesion that had a confirmed diagnosis of pancreatitis and one suspicious lung met was confirmed as a granulomatous infection. Even without this retrospective information, when there was agreement, the confidence level for PET/CT was 4.4/5.0 versus 4.0/5.0 for SPECT/CT. Importantly, as a result of these findings, the FDA agreed to the following important modifications to the Phase 3 protocol:

1. All certified SPECT/CT systems used in the Phase 3 study must have a CT that provides an AC reconstruction slice thickness of 5 mm or better.
2. All suspicious lesions identified by the SPECT and/or PET core laboratory readers must be confirmed by either a tissue diagnosis or evidence for contrast enhancement from a baseline diagnostic CT contrast (DCCT) study to eliminate the possibility of a false positive interpretation for ^18^F-FDG due to uptake in infection or inflammation.

**Figure 5:**
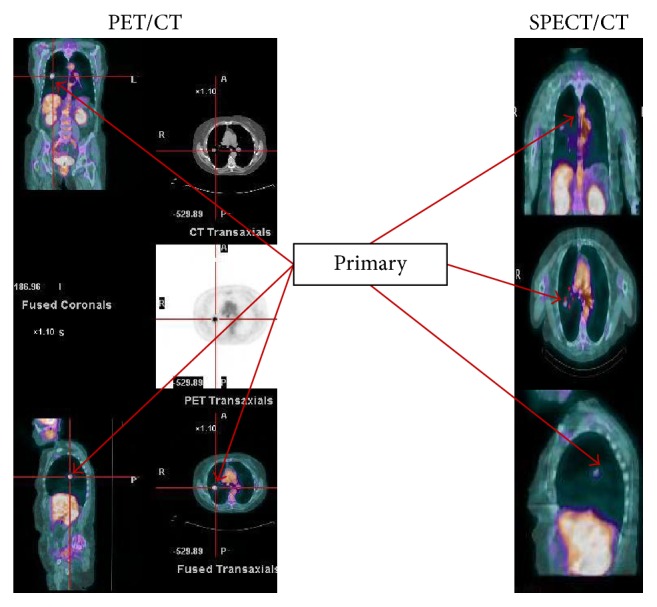
Comparative SPECT/CT: ^99m^Tc- EC-G and PET/CT: ^18^F- FDG image sets on a 68-year-old patient having a biopsy confirmed primary NSCLC.

**Figure 6:**
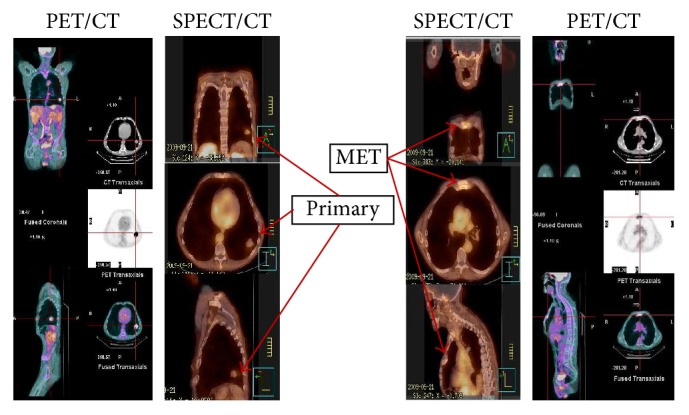
Comparative SPECT/CT: ^99m^Tc- EC-G and PET/CT: ^18^F- FDG image sets on a 56-year-old patient having a biopsy confirmed primary NSCLC and diagnostic CT contrast confirmed metastasis to the sternum.

**Figure 7:**
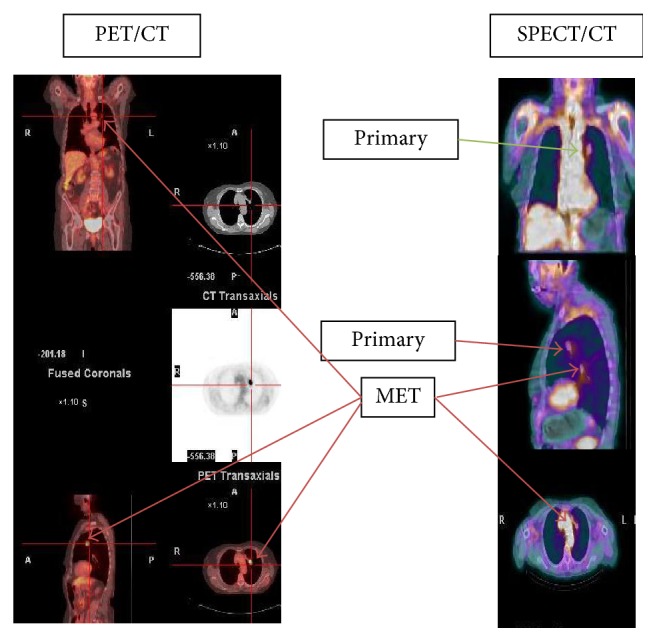
Comparative SPECT/CT: ^99m^Tc- EC-G and PET/CT: ^18^F- FDG image sets on a 61-year-old patient having a biopsy confirmed primary NSCLC and diagnostic CT contrast confirmed metastasis to the lung.

Example of image sets showing detection of primary tumors and metastasis for ^18^F-FDG PET/CT and ^99m^Tc-EC-G SPECT/CT is shown in Figures 5–7. In all cases the slice thickness for the reconstructed AC maps is 5 mm or better.

## 5. Discussion

The Phase 2 studies completed on 17 patients expanded the patient safety experience using ^99m^Tc-EC-G and provided evidence of ^99m^Tc-EC-G imaging efficacy in identifying anatomical regions with known non-small cell lung cancer and in determining the extent of disease (metastasis) in these patients as noninferior to ^18^F-FDG PET/CT imaging. Specifically, the Phase 2 study demonstrated equivalent detection (100%) of primary lesions (biopsied) by SPECT/CT and PET/CT (sensitivity only) and noninferiority to detectability (70% agreement) for metastatic lesions between SPECT/CT and PET/CT.

In addition, the Phase 2 study showed that ^99m^Tc-EC-G SPECT/CT imaging has the potential to serve as an alternative to ^18^F-FDG PET/CT imaging for diagnosing and staging oncology patients. In particular, the Phase 2 study provided equivalent diagnostic information with good accumulation in lung cancer and associated metastatic lesions using gamma cameras from three device manufacturers (Philips Healthcare, Siemens Healthcare, and GE Healthcare) as well as for a Philips SPECT system integrated to a Philips CT system via a Philips work station, provided the imaging device has a CT with 5 mm or better slice thickness for reconstructing AC maps and image fusion. The Phase 2 study also showed that when the specific activity of ^99m^Tc-EC-G was increased from 5 mg of EC-G labeled with 925 BCq of ^99m^Tc to 1 mg of EC-G labeled with 925 BCq of ^99m^Tc, the tumor/background ratio for both the primary and metastatic lesions improved significantly. It was also shown that when the time to image was changed from 2 to 3 hours after injection, there was additional improvement in the tumor/background ratio. Thus, the Phase 3 study design requires specific characteristics for SPECT/CT technology, high specific activity for ^99m^Tc-EC-G, and a 3-hour postinjection imaging time to assure the highest detectability of small lesions even in the presence of dense tissue.

An extremely important issue identified in Phase 2 relates to whether lesions detected by either SPECT or PET that do not have a tissue diagnosis are in fact malignant. For example, it is now known that 18F-FDG localizes in infection and inflammation as well as malignant tumors. The results of the Phase 2 study and the Study Design, Protocol and Statistical Analysis Plan for the pivotal Phase 3 study were presented to the FDA. The FDA granted the proposed Phase 3 study with a Special Protocol Assessment Letter of Agreement.

A summary of the main features of the Phase 3 study includes the following:

(1) The Phase 3 study was designed for noninferiority. Hypothesis testing of the results of the Phase 2 primary endpoint of sensitivity for detection of the biopsied primary lesion combined with the objective measure for detecting metastasis resulted in the following parameters being accepted by the FDA:

- An estimated 165–190 patients, for each of the two identical clinical arms
- A margin of 10% between SPECT/CT: 99mTc-EC-G and PET/CT: 18F–FDG
- A required confidence interval of 95%.

(2) The patient population was expanded to include all lung cancer types.

(3) Criteria for patient enrollment include any patient with clinical and radiological evidence consistent with lung cancer that has been referred for a PET scan to confirm the diagnosis and/or for staging the disease.

(4) All patients enrolled must agree to a tissue diagnosis of the primary lesion; a baseline diagnostic CT with contrast study; and a whole-body bone scan independent of the results of the PET/CT study.

(5) The Phase 3 study Truth Standard will be tissue diagnosis (primary as well as metastatic lesions when available) or evidence for contrast enhancement/or not from the diagnostic CT with contrast scan on suspicious lesions.

(6) The blinded read imaging interpretation by the Core Lab readers for the presence of malignancy for both PET and SPECT will be compared against the Truth Standard for every detectable lesion.

For this reason, Phase 3 will require that all suspicious lesions detected by either radiopharmaceutical compound be validated for malignancy. This will include a requirement that all primary lesions have a tissue diagnosis. In addition, a tissue diagnosis (open or closed biopsy) is performed whenever possible or practical in respect to any suspicious metastatic lesions. Suspicious metastatic lesions that do not have tissue confirmation will be deemed malignant if the baseline Standard of Care DCCT scan consensus interpretation by 2 independent truth standard core laboratory readers (readers who are independent/different from the core laboratory PET and SPECT readers) determines the lesions(s) to have vascular and anatomical patterns (contrast enhancement) consistent with malignancy. Phase 3 trial would categorize the patients into clinically statistical relevant groups such as percent of patients of NSCLC highlighting the need of 99mTc-EC-G for diagnostic tests to identify the subpopulations of patients who will benefit from this diagnostic/therapeutic trial.

## 6. Conclusions

^99m^Tc-EC-G was well tolerated. All but 2 AEs (nausea and vomiting, considered mild and unlikely related to the study drug) were considered unrelated to study drug. There were 6 SAEs, all considered unrelated to the study drug, and there were no deaths or AEs leading to withdrawal. No clinically significant trends or abnormalities were observed in vital sign measurements or EKGs.

The ^99m^Tc-EC-G SPECT/CT results compared to the ^18^F-FDG PET/CT results showed 100% agreement for detection of primary lesions, determination of location and lesion size, and confidence that the detected lesion represented a malignancy. Detection of primary lesions was not influenced by the dose of ^99m^Tc-EC-G or by the time of imaging in the range of doses and times used in the study. Detection of primary lesion was also not influenced by the type of imaging device or the device vendor. Overall, the study results demonstrated noninferiority in detecting primary lesions (biopsied) of ^99m^Tc-EC-G SPECT/CT compared to ^18^F-FDG PET/CT (sensitivity only). Metastatic lesions were shown to localize EC-G for both the 1 and 5 mg dose of EC-G, but the detectability of lesions by SPECT/CT was at a lower level than that noted for PET/CT when the CT slice thickness for reconstruction of AC maps and image fusion was 1 cm. Despite this issue, the overall detectability, lesion location and size, and confidence that a suspicious lesion was malignant showed overall noninferiority to the same measures reported for ^18^F-FDG PET/CT (4.4/5.0 versus 4.0/5.0), respectively. The results of the Phase 2 study were warranted for the pivotal Phase 3 study.
